# One Target, Different Results: The Clinical Impact of Diagnostic Kit Choice in *BCR*::*ABL1* Testing for Chronic Myeloid Leukemia

**DOI:** 10.3390/diagnostics16142216

**Published:** 2026-07-15

**Authors:** Mirjana Suver Stević, Vlatka Periša, Karla Vujičić, Saška Marczi, Jasminka Sinčić-Petričević, Danijela Mjeda

**Affiliations:** 1 University Hospital Center Osijek, Josipa Huttlera 4, HR-31000 Osijek, Croatia; 2 Faculty of Medicine, Josip Juraj Strossmayer University of Osijek, HR-31000 Osijek, Croatia; 3 Department of Biology, Josip Juraj Strossmayer University of Osijek, HR-31000 Osijek, Croatia

**Keywords:** laboratory proficiency testing, leukemia, myelogenous, chronic, BCR-ABL positive, quantitative polymerase chain reaction, reverse transcriptase polymerase chain reaction

## Abstract

**Background:** Quantitative PCR measurement of *BCR*::*ABL1* is essential for monitoring molecular response and detecting relapse in chronic myeloid leukemia (CML) patients. Given the availability of multiple commercial kits for cDNA synthesis and minimal residual disease assessment, analytical accuracy and reliability are critical. This study evaluated two commercial RT and qPCR kits for *BCR*::*ABL1* quantification and fusion transcript variant identification. **Methods:** Total RNA was isolated from peripheral blood, bone marrow, and external quality control samples from the UK NEQAS for Leucocyte Immunophenotyping program. cDNA synthesis was performed using two kits: AffinityScript (ASK) and RT Kit (RTK). *BCR*::*ABL1* transcript levels were determined using the ipsogen^®^ BCR-ABL1 Mbcr IS-MMR Kit (IPS) and the LightMix^®^ bcr-abl t(9;22) M/m/µ Kit (TMB). Results were compared with UK NEQAS LI reference data. Fusion transcript variants were analyzed using nested PCR and a commercial qPCR assay. **Results:** Substantial variability was observed between the TMB and IPS assays, with moderate, non-significant correlation and wide limits of agreement. Established discrepancies resulted in different classifications of molecular response, and IPS results showed better concordance with external quality assessment data. *ABL1* quantification revealed significantly higher copy numbers with the RTK compared to the ASK (*p* < 0.0001), enabling more reliable assessment of deep molecular responses. Statistical analyses indicated systematic and proportional bias between the methods. For variant detection, nested PCR demonstrated higher specificity, while the commercial assay showed limited discriminatory capability. **Conclusions:** Significant methodological differences may affect clinical interpretation, underscoring the importance of validation and standardization in CML molecular monitoring.

## 1. Introduction

Chronic myeloid leukemia (CML) is a myeloproliferative neoplasm characterized by the clonal transformation of a pluripotent hematopoietic stem cell. The disease is defined by a reciprocal translocation between chromosomes 9 and 22, resulting in the formation of the Philadelphia chromosome and the oncogenic *BCR*::*ABL1* fusion gene, which drives leukemogenesis through constitutive tyrosine kinase activity and enhanced cellular proliferation [1,2,3,4,5,6]. This fusion gene encodes a constitutively active tyrosine kinase that drives leukemogenesis and serves as the primary therapeutic target in CML. The introduction of tyrosine kinase inhibitors (TKIs) has dramatically improved the prognosis of patients with CML, enabling effective disease control and long-term survival [7,8,9]. In patients who respond to therapy, TKI treatment leads to a progressive reduction in *BCR*::*ABL1* transcript levels, which are routinely monitored using quantitative polymerase chain reaction (qPCR)-based assays. The depth of molecular response (MR3–MR5) is assessed at predefined time points according to established international guidelines and recommendations, providing essential information for treatment evaluation and clinical decision-making [10,11,12]. Accurate molecular monitoring is therefore a critical component of CML management. However, molecular diagnostics comprises multiple analytical steps, including the collection of bone marrow and peripheral blood samples, RNA extraction, identification of *BCR*::*ABL1* transcript variants at diagnosis, complementary DNA (cDNA) synthesis, and subsequent qPCR analysis. Given the complexity of this workflow, rigorous methodological validation and verification are required to ensure the reliability and reproducibility of diagnostic results. In this context, we performed a comparative evaluation of two commercial cDNA synthesis and qPCR kits, together with an in-house nested PCR method and a commercial PCR assay. Our findings underscore the importance of comprehensive validation before the implementation of molecular diagnostic methods in routine clinical practice. Inadequately validated assays may produce inaccurate results, lead to incorrect classification of molecular response depth, and ultimately affect clinical decision-making and patient management.

## 2. Materials and Methods

The study analyzed RNA samples from peripheral blood and bone marrow of CML patients monitored at the Department for HLA Typing and Genomic Diagnostics, University Hospital Center Osijek, Croatia. To compare the qPCR results and evaluate the unbiased reliability of the applied assay sets, the same assay sets were used to analyze samples obtained from UK NEQAS for Leucocyte Immunophenotyping (UK NEQAS LI) through participation in an external quality assessment (EQA) program. To ensure the reproducibility of the results, all applied assay sets were initially tested on ten diagnostic samples in two independent runs prior to the main study. The selection of samples for reproducibility testing was performed in such a way that each response category (MR1–MR5) was represented by two samples. Laboratory personnel performing the qPCR analyses were blinded to the results obtained with the alternative assay in order to minimize potential bias during data acquisition and interpretation. After testing the same selected samples in duplicate using both kits, Cp values were compared, with the requirement that the difference between replicates did not exceed one Cp value. After reproducibility had been confirmed, analyses were performed on all samples included in the study.

### 2.1. RNA Extraction

Peripheral blood leukocyte counts were measured using a Mindray BC-3600 analyzer (Mindray, Shenzhen, China), and RNA was extracted from 2 × 10^7^ mononuclear cells. The lyophilized samples obtained from UK NEQAS LI (Sheffield, UK) were processed using the same RNA extraction protocol based on the phenol-based TRI Reagent^®^ method (Sigma-Aldrich, Darmstadt, Germany). The concentration and purity of RNA were determined using a spectrophotometer (Molecular Devices Quick Drop, San Jose, CA, USA).

### 2.2. Complementary DNA Synthesis

cDNA was synthesized using two commercially available kits: the AffinityScript qPCR cDNA Synthesis Kit (Agilent Technologies, Santa Clara, CA, USA; Lot No. 0006709178; ASK) and the RT Kit (Qiagen, Venlo, The Netherlands; Lot No. 9780077; RTK). RNA samples were used at a concentration of 1 ng/µL for both procedures.

cDNA synthesis with the ASK was performed in one step under the following conditions: 25 °C for 5 min, 42 °C for 15 min, 95 °C for 5 min, and cooling to 4 °C.

For the RTK, synthesis was carried out in two steps. In the first step, RNA samples were incubated at 65 °C for 5 min, after which the reaction master mix was added. The second step was performed under the following conditions: 25 °C for 10 min, 50 °C for 60 min, 85 °C for 5 min, and cooling to 4 °C for 5 min.

To evaluate the reliability of the cDNA synthesis kits, qPCR was performed using primers specific for the control gene *ABL1* provided in IPS (Section 2.3). The *ABL1* copy number in samples synthesized by ASK and RTK was determined by relative quantification, using standards ranging from 10^3^ to 10^6^ copies.

### 2.3. Quantitative Analysis of BCR::ABL1

Relative quantification of the *BCR*::*ABL1* fusion transcript was performed using commercial kits from two different manufacturers: LightMix^®^ Kit bcr-abl t(9;22) M/m/µ and ABL1 Reference (TIB MolBiol, Berlin, Germany; Lot No. 27362101; Correction Factor 1.875; TMB) and ipsogen^®^ BCR-ABL1 Mbcr IS-MMR Kit (Qiagen, Venlo, The Netherlands; Lot No. 97503343, Correction Factor: 0.099 ± 0.05; IPS), on a real-time PCR instrument, LightCycler 480 II (Roche, Basel, Switzerland). The limit of detection (LOD) for both sets was set at 10 copies of *BCR*::*ABL1* per reaction, defined by the lowest standard (SP1), which must be detected in every run. Both kits were evaluated using identical cDNA samples synthesized by RTK. Reaction mixtures and qPCR cycling conditions were set according to the manufacturers’ instructions. Each kit included six standards covering a concentration range from 10^1^ to 10^6^ copies of the fusion transcript and the reference gene *ABL1*, which were used to determine the relative concentration of the *BCR*::*ABL1*. Both kits allow conversion of results to the International Scale (IS) using a lot-specific correction factor as well as a high-positive control (>1% *BCR*::*ABL1*). For both kits, separate reaction mixtures were prepared for the detection of the *BCR*::*ABL1* and the reference gene *ABL1*. qPCR conditions were defined according to the manufacturers’ protocols. The main difference between the applied kits lies in the spectrum of detectable fusion transcripts. The TMB kit detects and quantifies a broader panel of transcripts (M-, m-, and µ-bcr), e13a2, e13a3, e14a2, e14a3, e1a2, e1a3, e19a2, and e13a2, while IPS detects and quantifies the two most common variants: e13a2 and e14a2 (M-bcr). The obtained results were converted and expressed according to the IS. The depth of cytogenetic and molecular response was determined according to the recommendations of Cross et al. [13].

### 2.4. Characterization of BCR::ABL1 Fusion Transcript Variants

To determine *BCR*::*ABL1* fusion transcript variants and assess methodological reliability, results from the commercial PCR assay TMB were compared with those obtained using a BIOMED-1-recommended nested PCR approach [14].

The TMB allows identification of the fusion transcript type (M-bcr, m-bcr, and/or µ-bcr) without resolving the exact transcript variant. Sample preparation involved a reaction mixture with primers specific to the given fusion transcripts. PCR conditions were identical to those used for qPCR. Results were analyzed on filter combination 498–640, and fusion transcripts were identified on Cp values corresponding to a decrease in fluorescence signal, visualized as distinct peaks.

In parallel, fusion transcript variants were further characterized using a nested PCR, which enables precise identification of M-bcr variants (e13a2, e14a2, e13a3, e14a3) and m-bcr variants (e1a2, e1a3). Target sequences from cDNA were amplified in two consecutive PCR rounds using different primer sets on a Veriti PCR instrument (Applied Biosystems, Foster City, CA, USA). Primer sequences and PCR conditions were applied as described in the referenced protocol [14]. PCR products were separated by electrophoresis on a 2% agarose gel for 90 min at 250 V and 180 mA. Fusion transcript variants were assigned based on fragment size by comparison with a DNA size marker of known fragment length (OLERUP, Saltsjöbaden, Sweden).

### 2.5. Statistical Analysis

Statistical analyses were performed using MedCalc software (MedCalc Software Ltd., version 23.4.8., Ostend, Belgium, https://www.medcalc.org/en/calc/test_correlation.php, accessed on 3 February 2026). Continuous variables are presented as mean ± standard deviation (SD) or median with interquartile range (IQR), depending on data distribution. The normality of continuous variables was assessed using the Shapiro–Wilk test. For both assay comparisons (IPS vs. TMB and RTK vs. ASK), paired measurements were initially evaluated using descriptive statistics. Depending on data distribution, differences between paired measurements were assessed using either the paired Student’s *t*-test or the Wilcoxon signed-rank test. Pearson’s correlation coefficient and Spearman’s rank correlation coefficient were calculated to describe the strength of association between assays. However, correlation analyses were considered descriptive only and were not interpreted as measures of analytical agreement. Method agreement was primarily evaluated using Passing–Bablok regression. Regression slope and intercept, along with their corresponding 95% confidence intervals (CIs), were calculated. The cumulative sum (CUSUM) test was used to assess deviation from linearity. Agreement between assays was further evaluated using Bland–Altman analysis. Mean bias, 95% confidence intervals of the bias, and the 95% limits of agreement (LoA) together with their corresponding 95% confidence intervals were calculated. Because regression-based method comparison requires quantitative measurements from both assays, samples with undetectable *BCR::ABL1* transcript levels (value = 0 in either assay) were excluded from Passing–Bablok and Bland–Altman analyses. All statistical tests were two-sided, and a *p*-value < 0.05 was considered statistically significant.

## 3. Results

In all analyses, the predefined qPCR quality criteria were fulfilled. The standard curves were linear, with an R^2^ value of 0.999 and slopes ranging from −3.232 to −3.312. The difference in Cp values between technical replicates did not exceed one cycle in any analysis. Additionally, the lowest standard (SP1, 10 copies) was successfully quantified in all analyses.

### 3.1. Quantitative Analysis of BCR::ABL1

To evaluate the reliability of two commercially available kits for *BCR*::*ABL1* transcript quantification, 24 samples were analyzed in duplicate, including 16 samples from Philadelphia chromosome-positive CML patients (Table 1) and eight EQA samples provided by UK NEQAS LI. Three samples had undetectable *BCR::ABL1* transcript levels (value = 0 in one of the assays) and were therefore excluded from regression-based agreement analyses. Consequently, 21 paired measurements were included in the Passing–Bablok regression and Bland–Altman analyses. Passing–Bablok regression demonstrated no statistically significant constant or proportional bias between IPS and TMB. The estimated intercept was close to zero (0.0003), and its 95% confidence interval included the expected value of zero (−0.0507 to 0.0198), indicating the absence of constant bias. Likewise, the slope did not significantly differ from one (0.455), as its 95% confidence interval included the expected value (0.165 to 1.657), indicating no evidence of proportional bias. Furthermore, the CUSUM test showed no significant deviation from linearity (*p* = 0.787), confirming that the relationship between both assays was adequately described by a linear model. Although Passing–Bablok regression did not identify systematic bias, Bland–Altman analysis demonstrated considerable variability between assays. The mean bias was −4.591% IS (95% CI: −13.373 to 4.191), indicating that, on average, neither assay consistently produced higher values than the other. However, the limits of agreement ranged from −42.405% IS (95% CI: −57.658 to −27.151) to 33.22% IS (95% CI: 17.969 to 48.476), demonstrating substantial inter-assay variability across the investigated concentration range. Retrospective sensitivity analysis showed that 80% power, corresponding to a Type II error probability of β = 0.20, would be achieved only for a symmetric agreement boundary of approximately ±64.4 measurement units. Therefore, the study was insufficiently powered to demonstrate agreement within narrower, analytically meaningful limits, indicating that a larger sample size would be required. Correlation analysis demonstrated only a moderate association between the two assays. Because correlation coefficients quantify association rather than analytical agreement, they were interpreted only as descriptive measures and were not used for assessing the interchangeability of the assays.

**Figure 1:**
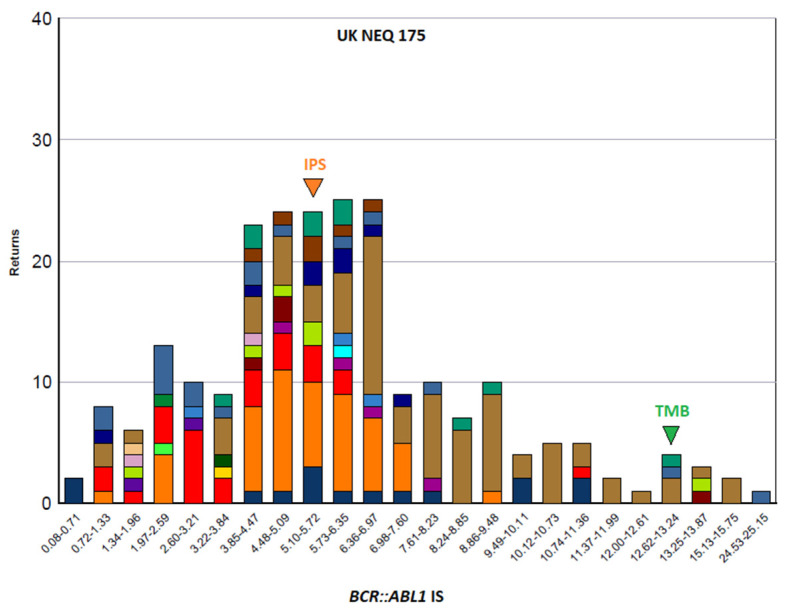

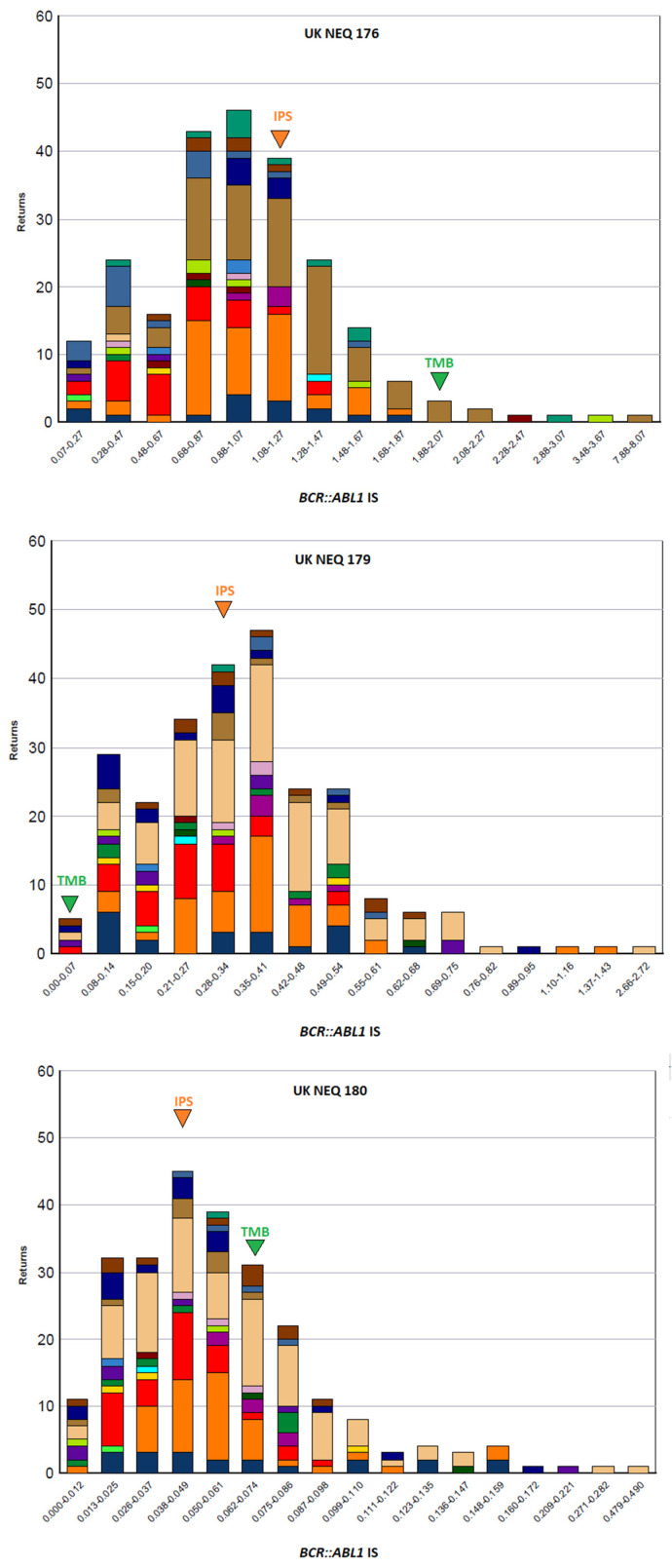

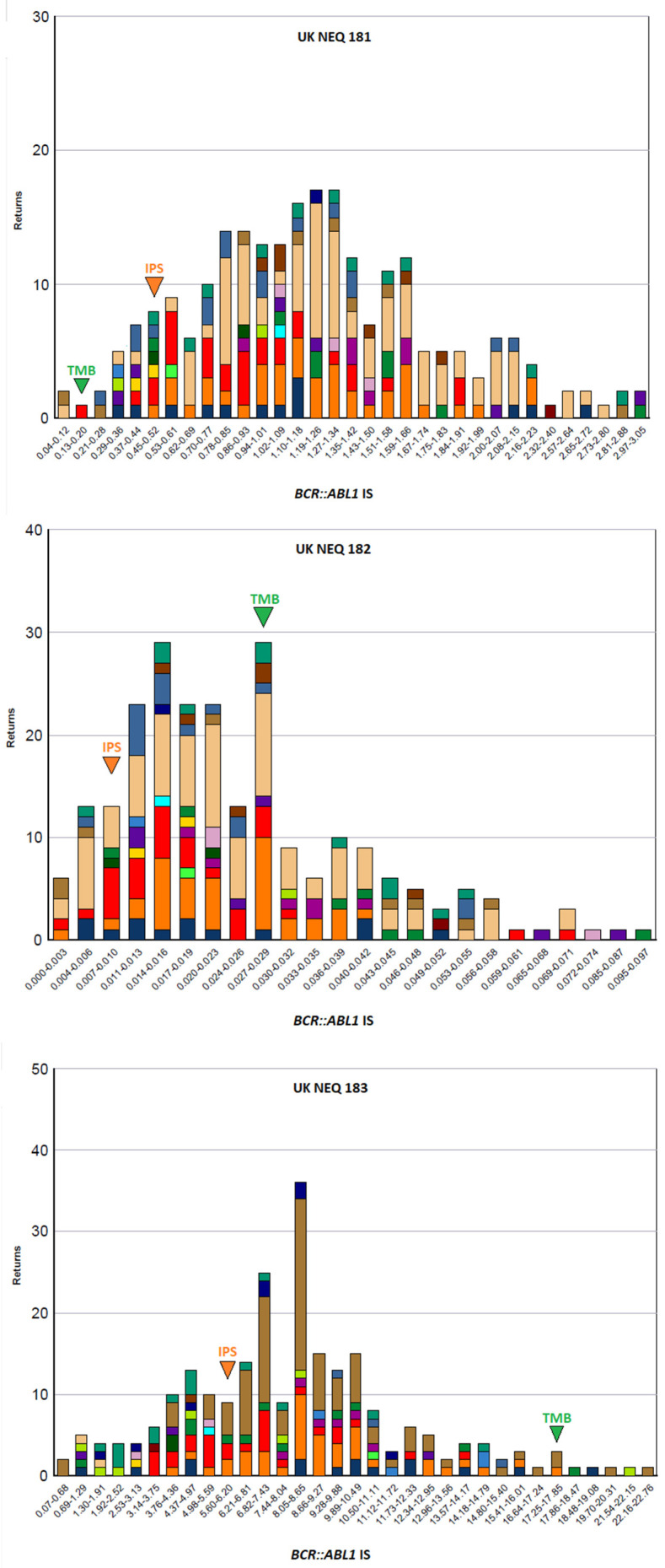

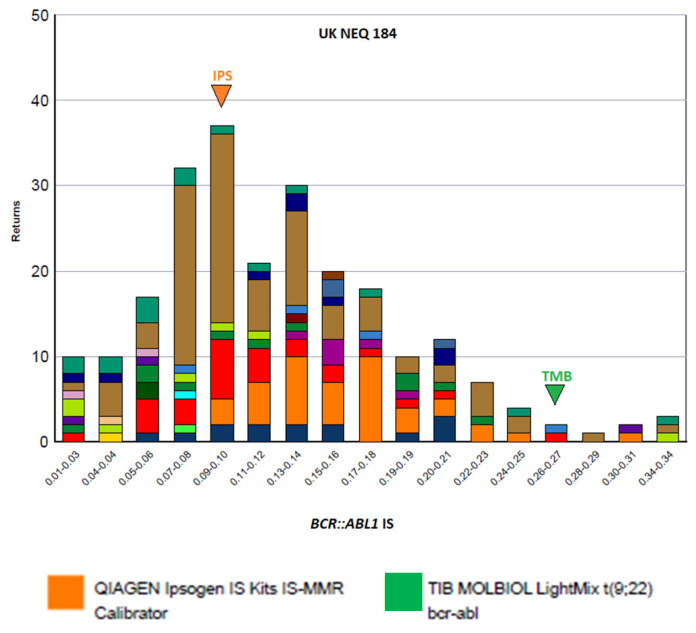
Comparison of results obtained using assay kits from two different manufacturers, LightMix^®^ Kit bcr-abl t(9;22) M/m/µ and ABL1 Reference (TIB MolBiol; TMB) and ipsogen^®^ BCR-ABL1 Mbcr IS-MMR Kit (Qiagen; IPS), using EQA samples provided by UK NEQAS LI. In the report, participants who used the IPS are indicated in orange, while those who used the TMB are shown in green. The orange and green arrows on the graphs indicate our results in relation to the results reported by laboratories participating in the EQA rounds. Permission to reproduce graphical images from the official *BCR*::*ABL1* major quantification EQA reports was granted by UK NEQAS LI.

Among the UK NEQAS LI samples, four showed higher transcript values with the TMB assay, two showed lower values, and two yielded comparable results. Concordant molecular response classifications were observed in three patient samples (MR3, MR4, and MR5) and one EQA sample (MR2), whereas most discrepant results differed by approximately one log level. Spearman’s rank correlation analysis demonstrated a moderate positive correlation between the assays (ρ = 0.434), although this was not statistically significant (*p* = 0.106). To further assess assay performance, results obtained with both kits were compared with UK NEQAS LI stratified data. The results of the IPS kit were generally located within the concentration ranges most frequently reported by participating laboratories, whereas those of the TMB kit often deviated from the consensus ranges. Although all submitted results achieved a ‘Satisfactory’ performance status, the greatest deviations for the IPS kit were observed in samples UKNEQ 181 and UKNEQ 182 (Figure 1). Overall, these findings indicate that, although IPS and TMB exhibit comparable average analytical performance, the observed inter-assay variability may influence molecular response classification in individual patients.

Overall, comparison with the EQA provider demonstrated that the IPS kit produced reliable results in our study. However, given the limited sample size, these findings should be interpreted with caution. The results should be confirmed in a larger cohort of samples before it can be concluded with a high degree of confidence that one of the two evaluated assays is more reliable for routine molecular monitoring of patients with CML.

### 3.2. Quantitative Analysis of ABL1

**Figure 2:**
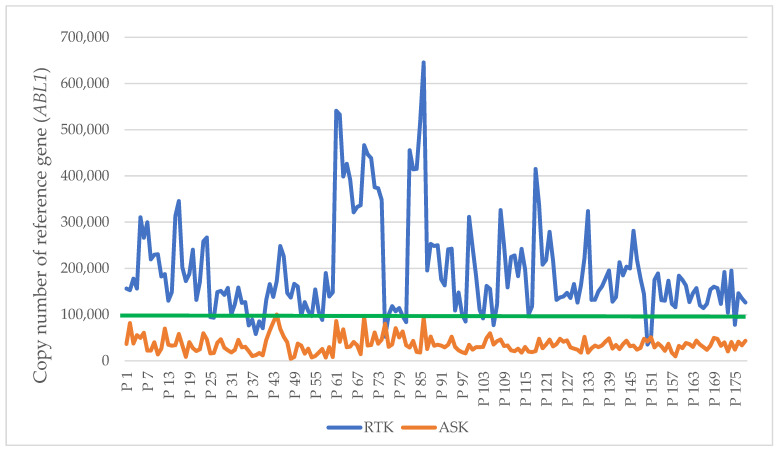
*ABL1* copy numbers obtained by qPCR after cDNA synthesis using RTK (blue line) and ASK (orange). The green line indicates the minimum amount of *ABL1* gene required to declare a deep molecular response (MR5).

A total of 178 RNA samples from patients with CML were analyzed. All cDNA samples synthesized using the RTK kit achieved the minimum *ABL1* copy number required for valid molecular response assessment, including thresholds for MR3 (≥10,000 copies) and MR4.5 (>32,000 copies) (Figure 2). Moreover, 158 samples (88.8%) exceeded 100,000 *ABL1* copies, enabling reliable evaluation of MR5. In contrast, none of the samples synthesized using the ASK kit reached 100,000 *ABL1* copies, while 76 samples (42.7%) failed to meet the MR4.5 threshold, and seven did not achieve the minimum requirement for valid molecular result reporting. RTK generated significantly higher *ABL1* copy numbers (194,547 ± 105,695) than ASK (35,131 ± 17,261) (*p* < 0.0001).

This difference was highly significant according to both the paired Student’s *t*-test (*p* < 0.001) and the Wilcoxon signed-rank test (*p* < 0.001). The higher *ABL1* copy numbers obtained with RTK had a direct impact on the assessment of molecular response. Although Pearson’s (*r* = 0.282) and Spearman’s (ρ = 0.258) correlation coefficients were statistically significant (*p* < 0.001), these analyses were considered descriptive only because of the association between correlation measures rather than agreement between analytical methods. On log10-transformed data, Bland–Altman analysis showed a mean bias of 0.742 log10 units (95% CI 0.702 to 0.783), corresponding to a geometric mean RTK/ASK ratio of 5.53. The 95% limits of agreement ranged from 0.210 to 1.275 log10 units, equivalent to ratio limits of approximately 1.62 to 18.82. Passing–Bablok regression on the log10 scale yielded the equation log10(RTK) = 4.010 + 0.266 × log10(ASK), with a slope 95% CI of 0.104 to 0.433 and an intercept 95% CI of 3.268 to 4.754. For the comparison of the RTK and ASK methods, the estimated statistical power was 80%, corresponding to a Type II error probability of β = 0.20. Overall, the RTK cDNA synthesis kit produced substantially higher *ABL1* copy numbers, enabling more reliable assessment of deep molecular responses in the majority of analyzed samples.

### 3.3. Determination of Fusion Transcripts

*BCR*::*ABL1* transcript variants were analyzed in 13 diagnostic RNA samples using nested PCR and the TMB qPCR assay. Nested PCR identified the e14a2 variant in 11 samples and the e13a2 variant in two samples. The TMB assay detected the M-bcr transcript in all e14a2-positive samples, consistent with the nested PCR findings. However, in the two samples classified as e13a2 by nested PCR, the TMB assay detected both M-bcr and m-bcr transcripts, whereas nested PCR identified only the e13a2 variant.

## 4. Discussion

Quantitative monitoring of the *BCR*::*ABL1* fusion transcript by qPCR represents a cornerstone for assessing and tracking therapeutic response in patients with CML. Given that critical clinical decisions—such as evaluation of molecular response depth and consideration of TKI discontinuation—are based on precise transcript quantification, it is essential that the diagnostic methods applied ensure a high level of analytical reliability. This study indicates that different kits may yield substantially divergent copy number values of the fusion transcript, which could, in turn, significantly impact clinical management [15,16]. Additionally, the results emphasize the critical importance of assay validation and result standardization prior to implementation in routine diagnostics [17,18]. In most diagnostic samples, the observed differences corresponded to approximately one logarithmic level, which is clinically relevant because molecular response categories are defined by log reductions in transcript levels. The most pronounced discrepancy was observed in sample S1, where the TMB assay indicated an MR2 response, whereas the IPS assay showed an undetectable transcript level corresponding to a deep molecular response (MR5). Such differences may have significant clinical implications, particularly in the context of evaluating treatment efficacy or considering treatment discontinuation in patients with sustained undetectable transcript levels [19]. Moreover, falsely elevated results suggesting loss of molecular response (e.g., samples S11, S15, and S16) could lead to incorrect conclusions regarding disease relapse or the development of therapeutic resistance, potentially prompting unnecessary transition to later lines of therapy [12]. Marked differences between the two assays were observed in samples S15 (IPS 48.90% vs. TMB 0.0004%) and S16 (IPS 64.69% vs. TMB 0.0138%), with variability in estimated *BCR*::*ABL1* transcript levels depending on the method used (Table 1). Sample S15 represented a newly diagnosed CML case prior to TKI therapy, while sample S16 originated from a patient with imatinib and nilotinib resistance associated with *ABL1* kinase domain mutations (L248V and L248_274del), consistent with high residual disease. Comparable results for S16 given by IPS were confirmed in an independent laboratory, as the patient was followed in two clinical centers with separate diagnostic facilities. In both cases, lower values were obtained using the TMB assay, leading to differences in the estimated depth of the molecular response. These discrepancies likely reflect methodological variability between assays and highlight the importance of assay selection in result interpretation. Although pre-analytical factors cannot be fully excluded, adequate *ABL1* copy numbers were observed. Repeat testing of discrepant samples may be considered to confirm the consistency of results. The reliability of IPS-derived results was confirmed by comparison with UK NEQAS LI EQA data, where most IPS results fell within concentration ranges reported by the majority of participating laboratories, achieving a “*Satisfactory*” performance status. In contrast, TMB-derived results more frequently deviated from consensus ranges and would be classified as “*Critical*.” Given that the comparative study for *BCR*::*ABL1* fusion transcript quantification was conducted on a limited number of diagnostic samples and the obtained results demonstrated considerable heterogeneity, it is important to emphasize that the study should be expanded using a larger sample cohort. Therefore, the current findings are not sufficient to support definitive conclusions regarding the suitability or unsuitability of either assay for routine diagnostic use. Furthermore, recent evidence increasingly links hematologic malignancies and treatment response to the gut microbiome, suggesting that it may contribute to biological variability in hematologic cancers, including chronic myeloid leukemia, through effects on immune regulation, inflammation, and drug metabolism [20,21]. Although these are not investigated in the present study, such factors may represent an additional source of heterogeneity in treatment response and disease monitoring among patients receiving TKIs. Following the publication of the 2023 European LeukemiaNet laboratory recommendations, several studies have further confirmed the analytical performance of standardized commercial qPCR assays for *BCR*::*ABL1* monitoring. Hyerin et al. demonstrated excellent agreement between the newly developed 1-copy *BCR*::*ABL1* qPCR assay and the QIAGEN ipsogen *BCR*::*ABL1* Mbcr IS-MMR assay, with high concordance at clinically relevant molecular response thresholds, supporting the reliability of standardized commercial assays in routine clinical practice [22]. Similarly, recent evaluations of automated cartridge-based platforms, including the GeneXpert *BCR*::*ABL1* Ultra system, have reported high reproducibility, excellent correlation with established qPCR methods, and reliable quantification across clinically relevant *BCR*::*ABL1* levels [23]. These findings are consistent with our results, in which the Odity and Ipsogen assays demonstrated excellent quantitative correlation. However, as emphasized by the Bland–Altman analysis in our study, correlation alone does not establish interchangeability. The observed limits of agreement indicate that the two assays should not be used interchangeably for longitudinal monitoring without prior analytical validation and consideration of clinically acceptable differences, particularly when monitoring deep molecular responses where small quantitative differences may influence clinical interpretation.

Regarding the reference gene, accurate quantification represents a fundamental prerequisite for reliable molecular monitoring in CML. The number of reference gene copies directly determines both the sensitivity of *BCR*::*ABL1* detection and the ability to assess deep molecular responses [24,25]. According to international recommendations, a minimum number of reference gene copies is required for reliable interpretation of molecular response levels [7,26]. Our results revealed a marked difference in *ABL1* copy number, with the RTK consistently generating substantially higher values than the ASK. This difference was statistically significant and suggests more efficient reverse transcriptase activity, which plays a crucial role in qPCR performance, particularly at very low transcript levels. The higher *ABL1* copy numbers obtained with the RTK enabled reliable assessment of both major (MR3, MR4) and deep molecular responses (MR4.5, MR5) [1,4]. In contrast, cDNA synthesis using the ASK resulted in significantly lower *ABL1* copy numbers, with a considerable proportion of samples failing to reach thresholds required for assessment of deeper molecular responses [10,26,27]. None of the analyzed samples reached the 100,000-copy threshold required for MR5 evaluation [10,12], and in many samples, MR4.5 could not be determined due to failure to achieve the 32,000-copy threshold. Additionally, several samples did not meet the minimum requirement of 10,000 *ABL1* copies necessary for reporting a valid molecular result, thereby precluding reliable clinical interpretation and necessitating repeat sampling in routine practice [13]. These differences may be attributed to variations in enzyme properties, reaction buffer composition, or primer design. Additionally, differences in reaction conditions—particularly the duration of cDNA synthesis—may contribute to the observed variability, as the ASK protocol requires less than 30 min compared to approximately 2.5 h for the RTK protocol, potentially limiting enzyme efficiency. Furthermore, the RTK protocol includes an initial RNA denaturation step, which improves the accessibility of RNA for primer binding and reverse transcription, whereas this step is absent in the ASK protocol. It is also noteworthy that the TMB manufacturer recommends a different cDNA synthesis kit (Transcriptor First Strand cDNA Synthesis Kit, Roche, Basel, Switzerland). In preliminary experiments, results obtained with the recommended kit were consistent with those generated using the RTK.

In addition to qPCR-based monitoring and reference gene quantification, discrepancies were also observed in the methods for determining *BCR*::*ABL1* transcript variants. According to the literature and our experience, approximately 95% of CML patients harbor major *BCR*::*ABL1* transcript variants (e13a2 or e14a2), while only 1–2% present with minor transcripts (m-bcr). Furthermore, specific transcript variants may influence the kinetics of molecular response to TKI therapy, and several studies have shown that patients with the e14a2 more frequently achieve faster and deeper molecular responses [28,29,30,31,32]. This phenomenon may arise from alternative splicing or clonal evolution of leukemic cells and has been linked to disease progression or TKI resistance, without significantly impacting long-term prognosis or overall survival [33,34]. In our analysis, the TMB assay indicated the presence of both M-bcr and m-bcr transcripts in two samples, whereas nested PCR detected only the e13a2 variant (M-bcr). Given that simultaneous presence of both transcript types is rarely reported in CML, and considering the design limitations of the TMB assay, this finding most likely reflects an analytical artifact resulting in false-positive detection of the m-bcr transcript [35,36]. Unlike nested PCR, the exact binding sites of primers and probes in the TMB assay are not fully disclosed, precluding definitive conclusions regarding the origin of these results. In conclusion, nested PCR represents a more reliable method for identifying specific *BCR*::*ABL1* variants due to its use of variant-specific primers targeting well-defined breakpoint regions [14]. While commercial TMB qPCR assays offer faster processing, their ability to precisely identify individual variants may be limited.

Finally, this study has several limitations, primarily related to the relatively small sample size. Additionally, the quantification of reference genes focused exclusively on *ABL1* without evaluating its downstream impact on *BCR*::*ABL1*. Regarding transcript variant detection, the current findings do not allow definitive confirmation or exclusion of the simultaneous presence of major and minor transcripts. Although the m-bcr variant is more characteristic of ALL, it has been reported in a small subset of CML patients, occasionally in combination with major transcripts, particularly in blast crisis [25,33,37,38]. Independent validation using EQA samples would be necessary to confirm these findings.

## 5. Conclusions

Significant inter-assay variability was observed between the commercially available kits evaluated for *BCR*::*ABL1* quantification and cDNA synthesis, indicating that assay selection may influence the interpretation of molecular response in patients with chronic myeloid leukemia. Under the conditions of this study, the IPS assay showed better agreement with the EQA consensus results than the TMB assay. While differences in agreement with EQA results and *ABL1* copy numbers were observed between the evaluated assays, these findings should be interpreted with caution and do not establish the superiority of one platform over another. Nested PCR proved to be a reliable method for the identification of *BCR*::*ABL1* transcript variants. Overall, the results highlight the importance of careful assay selection, methodological standardization, and participation in EQA programs for accurate molecular monitoring of CML. Further studies involving larger cohorts and comprehensive analytical validation are warranted to confirm these findings.

## Figures and Tables

**Table 1:** Comparison of *BCR*::*ABL1* (IS) transcript levels in patient samples monitored during tyrosine kinase inhibitor therapy and UK NEQAS LI EQA samples tested using diagnostic kits from two different manufacturers (TMB and IPS). Diagnostic samples (**A**) and UK NEQAS LI samples (**B**) are stratified according to detected molecular response (MR3–MR5) and with *BCR*::*ABL1* transcript levels > 1%. (**A**) Comparison of *BCR*::*ABL1* transcript quantification results obtained with the IPS and TMB assays, together with the demographic characteristics of the patients included in the study. (**B**) presents, in addition to a comparison of the obtained values, the z-score analysis and performance status for the analyzed samples. For UK NEQAS LI scored samples, a ‘*Satisfactory*’ performance status is defined as a z-score between 2.0 and −2.0, and an ‘action’ performance status is defined as a z-score between 2.0 and 3.0 or −2.0 and −3.0. A z-score result above 3.0 or below −3.0 is considered a ‘*Critical*’ performance status.

**(A)**							
**Detected Molecular Response in Diagnostic Samples (%)**							
**Patient ID**	**Age**	**Sex**	**Disease Phase**	**Treatment Status**	**TKI Therapy**	**TMB**	**IPS**
S1	49	F	Chronic Phase	TKI	imatinib	0.6822	0
S2	45	F	Chronic Phase	TKI	dasatinib	0.0008	0
S3	71	M	Chronic Phase	TKI	nilotinib	0.0202	0.0025
S4	71	F	Chronic Phase	TKI	nilotinib	0	0.0043
S5	79	F	Chronic Phase	TKI	imatinib	0.0018	0.0034
S6	53	M	Chronic Phase	TKI	imatinib	0.0099	0.0062
S7	37	M	Chronic Phase	TKI	imatinib	0.0040	0.0119
S8	37	M	Chronic Phase	TKI	imatinib	0.0072	0.0270
S9	68	M	Chronic Phase	TKI	imatinib	0.0774	0.0775
S10	72	M	Chronic Phase	TKI	imatinib	0.1416	0.0318
**% of *BCR*::*ABL1* > 1% in diagnostic samples**							
**Patient ID**	**Age**	**Sex**	**Disease Phase**	**Treatment Status**	**TKI Therapy**	**TMB**	**IPS**
S11	74	M	Chronic Phase	TKI	imatinib	1.65	0.5125
S12	61	F	Chronic Phase	TKI	imatinib	8,17	23.52
S13	61	M	Chronic Phase	TKI	imatinib	18.14	29.57
S14	30	M	Chronic Phase	Newly Diagnosed	no treatment	33.01	5.45
S15	62	M	Chronic Phase	Newly Diagnosed	no treatment	48.90	0.0004
S16	70	F	Chronic Phase	TKI-resistant	ponatinib	64.69	0.0138
**(B)**							
**Detected Molecular Response in UK NEQAS LI Samples (%)**							
**Sample**		**TMB**	**z-Score**	**Performance Status**	**IPS**	**z-Score**	**Performance Status**
UK NEQ 179		0.0722	−2.64	Action	0.3375	−0.04	Satisfactory
UK NEQ 180		0.0067	−3.56	Critical	0.0468	−0.04	Satisfactory
UK NEQ 181		0.1988	−3.19	Critical	0.4584	−1.68	Satisfactory
UK NEQ 182		0.0273	−6.80	Critical	0.0093	−1.51	Satisfactory
UK NEQ 184		0.2600	1.47	Satisfactory	0.0929	−0.38	Satisfactory
**% of *BCR*::*ABL1* > 1% in UK NEQAS LI samples**							
**Sample**		**TMB**	**z-score**	**Performance Status**	**IPS**	**z-score**	**Performance Status**
UK NEQ 175		12.81	1.59	Satisfactory	5.27	−0.04	Satisfactory
UK NEQ 176		1.92	1.34	Satisfactory	1.2	0.50	Satisfactory
UK NEQ 183		17.31	1.43	Satisfactory	5.99	−1.13	Satisfactory

## Data Availability

The data presented in this study are available on request from the corresponding author due to privacy and ethical restrictions.
